# Intratympanic steroid treatments rescued recurrent hearing loss following COVID-19 vaccination and detection of an intralabyrinthine schwannoma

**DOI:** 10.1136/bcr-2022-249316

**Published:** 2022-07-06

**Authors:** Weihang Li, Alan Micco, Deyu Fang, Huiping Liu

**Affiliations:** 1 Aeronautical & Astronautical Engineering, Purdue University, West Lafayette, Indiana, USA; 2 Otolaryngology, Northwestern University Feinberg School of Medicine, Chicago, Illinois, USA; 3 Pathology, Northwestern University Feinberg School of Medicine, Chicago, Illinois, USA; 4 Pharmacology, Northwestern University Feinberg School of Medicine, Chicago, Illinois, USA

**Keywords:** Ear, nose and throat/otolaryngology, Vaccination/immunisation, Oncology

## Abstract

It remains unclear how to effectively treat rare cases of sudden and recurrent hearing losses which might coincidently follow vaccination. We report the first case, to our knowledge, of systemic and local steroid administration to successfully treat sudden and recurrent left-ear hearing loss, respectively, following a second dose of the BNT162b2 COVID-19 mRNA vaccination which inflammatory response potentially affected an existing left intralabyrinthine schwannoma in a young male patient. This case highlights the importance and timing of intratympanic steroid treatment strategies to suppress the progressive symptoms and restore hearing to a stable condition, and therefore avoid permanent hearing loss which would otherwise demand a surgical removal of the schwannoma to improve vertigo and reconstitute artificial hearing.

## Background

To combat the COVID-19 pandemic, mRNA vaccines have been widely administered as one of the most effective approaches to protect from SARS-CoV-2 infections and/or severe disease.^1 2^ The adverse effects of mRNA vaccinations are mostly reported mild and temporary, such as short-term, mild-to-moderate pain at the injection site, fatigue and headache without requiring specific treatments.^2^ However, it has been reported that these vaccinations are possibly associated with 147 cases with sudden hearing loss, deafness, deafness unilateral, deafness neurosensory and hypoacusis,^3^ and incidence estimates of sudden sensorineural hearing loss after COVID-19 vaccination ranged from 0.3 to 4.1 per 100 000 per year.^3^ It was unclear how to specifically treat such sudden hearing loss. Here, we report a case of sudden and recurrent hearing loss following COVID-19 mRNA vaccination in coincidence with detection of intralabyrinthine schwannoma and subsequent improvement on oral and then intratympanic steroid treatments.

## Case presentation

**Figure 1:**
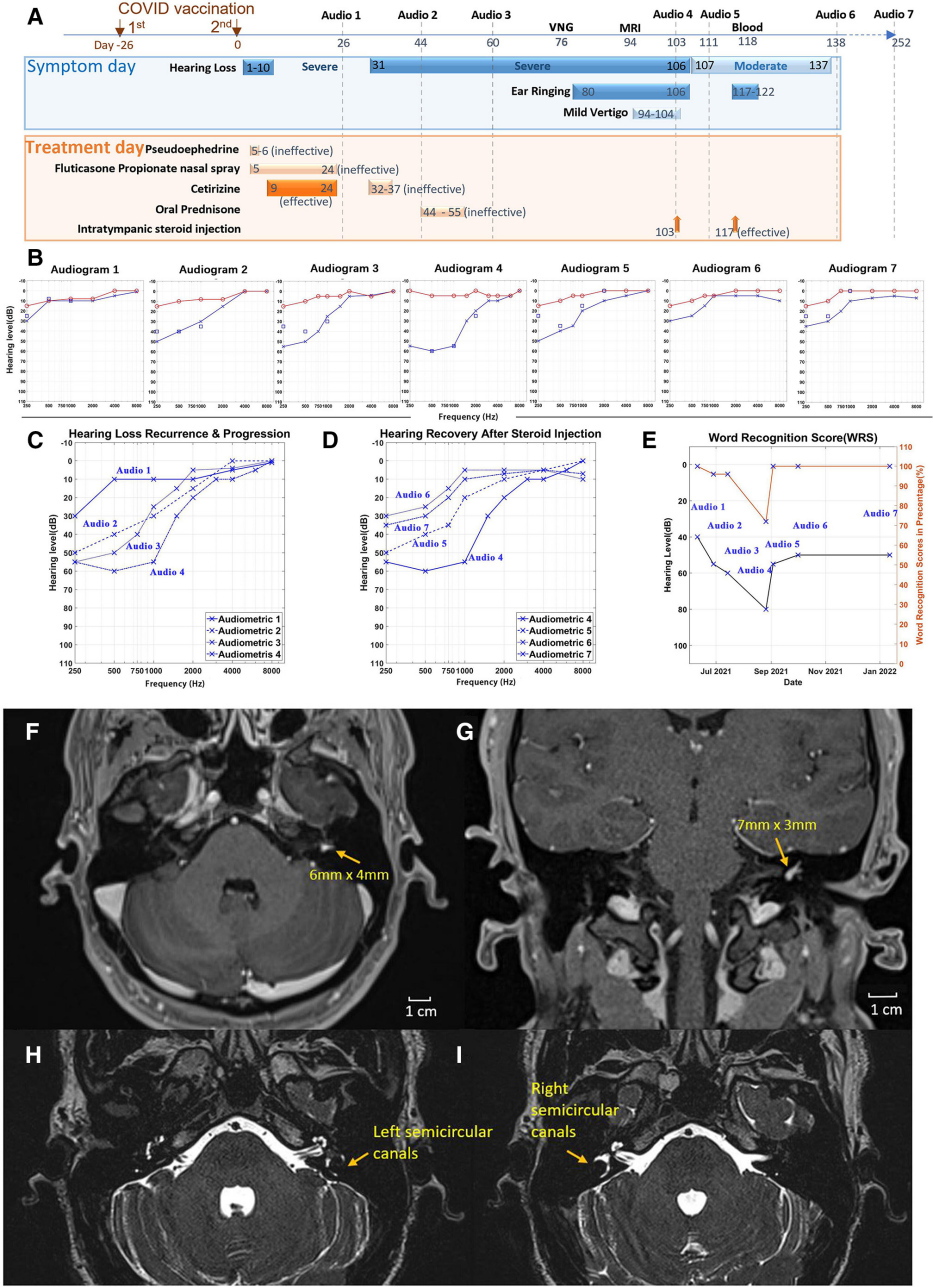
The timeline of clinical symptoms and treatments, audiometric and hearing measurement data, and MRI images of intralabyrinthine schwannoma. (A) The timeline for the two-dose COVID-19 vaccination (4 weeks apart); emerging symptoms of the first 10-day hearing loss starting on day 1 after the second dose of the BNT162b2 mRNA vaccine (day 0), the second (recurrent) hearing loss on day 31 lasting for 3.5 months along with ear ringing and mild vertigo; clinical tests (7 audiometric tests, MRI and blood draw), and the ineffective and effective treatments, including pseudoephedrine (ineffective), fluticasone propionate nasal spray (ineffective), first effective and second ineffective cetirizine treatments, ineffective oral prednisone, and final effective intratympanic steroid injections. No pure tone average abnormality or hearing loss symptoms were reported before vaccination. (B) Standard pure tone audiograms of seven sequential hearting tests at the indicated times in (A) for both right ear (red line) and left ear (blue line). Red circle represents air conduction. Blue square represents bone conduction and blue cross represents air conduction. (C) The accumulative audiogram of four left ear audiometric tests (1–4) showing the second hearing loss progression over 2 months. (D) The accumulative audiogram of four left ear audiometric tests (4–7) showing hearing recovery and stability after local steroid treatments to the left ear. (E) Left ear hearing level (air conduction) and word recognition score (WRS) in percentage (%) at the longitudinal series of 7 tests before and after intratympanic steroid injections. (F–I) Gadolinium-enhanced MRI of axial contrast-enhanced (F) and sagittal contrast-enhanced (G) volumetric T1w images with an intralabyrinthine schwannoma (arrow-pointed) in the left ear, and axial contrast-enhanced volumetric T2w images with the left semicircular canals (blurry) (H) and with the right semicircular canals (clear) (I).

A man in his 20s with normal vital records and blood pressure was referred to the ear, nose and throat (ENT) clinic for two times of sudden hearing losses of the left ear within three months following the second dose of COVID-19 vaccination. The oral steroids became ineffective for the recurrent hearing loss after initial effects to improve from the first hearing loss (figure 1A). He experienced the progressive hearing loss along with a continuous ringing and buzzing sound in the left ear, a slight spontaneous vertigo at the later stage of recurrent hearing loss, but no position-evoked vertigo was reported.

## Investigations

Based on the patient’s report, we tracked back the timeline of his symptoms, tests, and treatment trials and found a possible association with his COVID-19 mRNA vaccination, the second dose of which was administered on day 0 prior to his first sudden hearing loss of the left ear on day 1 (figure 1A). Subsequent treatments with pseudoephedrine and fluticasone propionate nasal spray for suspected allergic otitis media with effusion did not show any improvement. Therefore, to improve ear congestion before the first audio test, the patient took antiallergic cetirizine HCI orally at a dose of 10 mg every 24 hours for 2 weeks (day 9–24) that restored the patient’s left ear hearing almost back to normal based on patient’s self-report and the first audiometric test on day 26 (figure 1B, Audiogram 1), in which the tympanogram exhibited a normal bilateral type A pattern and masked bone conduction.

Unfortunately, 1 week later starting on day 31, the patient suffered from a second left-ear hearing loss with no response to the immediately resumed, 5-day cetirizine treatment, leading to impaired left ear hearing from most of the frequency range by day 44 (figure 1B, Audiogram 2). For a differential diagnosis of Meniere’s disease which might be associated with fluctuating hearing losses, a high dose of oral steroid treatment of prednisone (60 mg/day for day 44–50 and tapered off through 5 days) was given but ineffective to improve. The third audiometric test on day 60 indicated a progressive sensorineural hearing loss of the left ear for low frequency compared with the normal right ear (figure 1B–D, Audiograms 2 and 3), with a marginal decrease in word discrimination (%) (figure 1E), using the W-22 wordlist in the audio tests.

The patient was referred for videonystagmography test on day 76 which showed that the left ear had 93% weakness in the caloric testing and 50% directional preponderance to the right (online supplemental figure 1). Tracking and optokinetic tests displayed asymmetrical optokinetics and decreased intensity for the left side compared with the right side (online supplemental figures 2; 3).

10.1136/bcr-2022-249316.supp1 Supplementary data

10.1136/bcr-2022-249316.supp2 Supplementary data

10.1136/bcr-2022-249316.supp3 Supplementary data

Considering Meniere’s disease does not normally present with sudden hearing loss neither progressive hearing loss after oral steroid treatment, we hypothesised that an inflammatory response might be triggered by the second dose of COVID-19 mRNA vaccination and associated with two times of hearing losses. However, additional factors associated with the hearing loss needed to be determined.

## Differential diagnosis

A gadolinium-enhanced MRI was then performed at ENT on day 94, identifying a mass of about 6 × 4 mm labyrinthine schwannoma in the left vestibule (figure 1F, G). The images also revealed a reduction in the left side endolymph fluid (figure 1H, I). Thus, an internal labyrinthine schwannoma in the left side vestibule with extension into both the superior and lateral semicircular canals was detected.

The patient showed progressive left ear hearing loss on day 103 (figure 1B–E, Audiogram 4) along with left ear ringing and mild vertigo (figure 1A). To identify potential risk factors associated with hearing loss, additional blood tests were performed showing normal complete blood counts (with differential analyses) and normal rheumatoid factor results without detection of autoimmune response. The comprehensive metabolic panel and blood lipid laboratory tests were also normal, largely excluding the possibility of metabolic dysregulation caused hearing damage. Anti-SARS-CoV-2 antibodies were positively detected after vaccination (online supplemental figure 4).

10.1136/bcr-2022-249316.supp4 Supplementary data

## Treatment

Suspecting an inflammatory response against the labyrinthine schwannoma following the two-dose vaccination, an intratympanic steroid injection of dexamethasone (10 mg/mL) was given once on day 103 by the ENT doctor to completely fill the left middle ear. Three days after the first local steroid treatment (day 106), the patient reported disappearance of the left ear ringing and buzzing. The hearing test revealed an average of 20 dB improvement in the left ear hearing on day 111 (figure 1B–E, Audiogram 5). The patient then received the second intratympanic injection of 10 mg/mL dexamethasone to the left middle ear on day 117, resulting in a significant hearing recovery and word recognition score back to normal, shown by a hearing test on day 138 (figure 1B–E, Audiogram 6).

## Outcome and follow-up

Since the second treatment of local steroid injection on day 138, the patient had not reported any vertigo or further hearing loss for the following 4 months. The 7th audio test on day 252 showed a stable result similar as Audio 6 verifying the long-term improvement on the hearing (figure 1B–E, Audiogram 7).

## Discussion

The patient had no previous MRI scanning record nor hearing disorder history prior to COVID-19 vaccination. Considering that intralabyrinthine schwannoma would normally take years to develop without any symptoms, it most likely had developed before vaccination in this young patient. The fact that treatment with oral and intratympanic anti-inflammatory steroid medications to treat sudden and recurrent hearing loss implicates a possible immune response triggered to the pre-existing intralabyrinthine schwannoma following COVID-19 vaccination. However, there is no conclusive proof of causation between the vaccination and hearing loss.

A systematic review on auditory complications estimated a 7.6% of COVID-19 patients experienced with hearing loss, 14.8% with tinnitus and 7.2% with vertigo.^4^ An earlier report of several Iranian patients experienced hearing loss in one ear, as well as vertigo.^5^ Another case study reported an Egyptian man developed sudden hearing loss without any other coronavirus symptoms was tested positive for coronavirus.^6^ Further research is necessary to determine if and/or how the SARS-CoV-2 infection^7^ or COVID-19 vaccination induces acute otitis or hearing loss. In addition to other common clinical symptoms of respiratory virus infection (cough, fever, headache, loss of taste or smell and sore throat, etc), a variety of relatively rare clinical symptoms, including skin changes, eye problems, severe confusion (delirium) and sudden hearing loss are possibly increasing.^5 6^

While the American Academy of Otolaryngology Head and Neck Surgery guidelines for sudden hearing loss (below) need to be followed, this case demonstrates that when oral steroids lose effects on persistent and progressive hearing loss, advanced examination for possible organic diseases as well as timely and intensive immune suppression via intratympanic injections to the local microenvironment are necessary for a full recovery. It is critical for young patients to prevent permanent hearing loss and avoid possible surgical interventions. These data also suggest that vaccinated patients who suffer from inflammatory reactions and adverse effects should seek cautious evaluations, close symptomatic monitoring and strategic immune-suppressive treatment.

### The American Academy of Otolaryngology Head and Neck Surgery guidelines for sudden hearing loss

1. Clinicians should distinguish sensorineural hearing loss from conductive hearing loss. A sudden hearing loss is defined as a loss that occurs within a 72-hour period.
2. Audiometric evaluation should be obtained within the first 2 weeks of the occurrence to prove the hearing loss.
3. MRI is obtained to rule out retrocochlear involvement.
4. The patient is offered corticosteroid, either oral or intratympanic.

**Patient’s perspective**

When I first heard I had a tumour in the left ear, I was completely shocked and started to search online for any possible explanation and treatment. There was no such information available, so I felt helpless. I had prepared for permanent loss of my left ear hearing. After experiencing all the up and downs along with all the tests and treatment trials, I am extremely grateful for the final improvements. The submission and ultimate publication of this case report will absolutely help similar patients in the future.

**Learning points**

- This is a rare case of two sudden, recurrent progressive hearing losses which have immediately followed the second dose of COVID-19 vaccination and coincided with detection of labyrinthine schwannoma.
- Effective communications between the patient and a multidisciplinary team including otolaryngologist, virologists and cancer immunologists for differential diagnosis with MRI are pivotal to identify the association with COVID-19 vaccination and schwannoma.
- Following the ear, nose and throat guidelines, implementation of the intratympanic steroid injection approach is effective to mitigate the local aggressive inflammatory response and ultimately restore the hearing.
