# Supplementary material for: Intratympanic steroid treatments rescued recurrent hearing loss following COVID-19 vaccination and detection of an intralabyrinthine schwannoma
Source: BMJ Case Rep. 2022 Jul 6;15(7):e249316. doi: 10.1136/bcr-2022-249316 (PMC9260791; doi:10.1136/bcr-2022-249316)
Supplement: Supplementary data [file bcr-2022-249316supp002.pdf]

Supplementary Figure 2

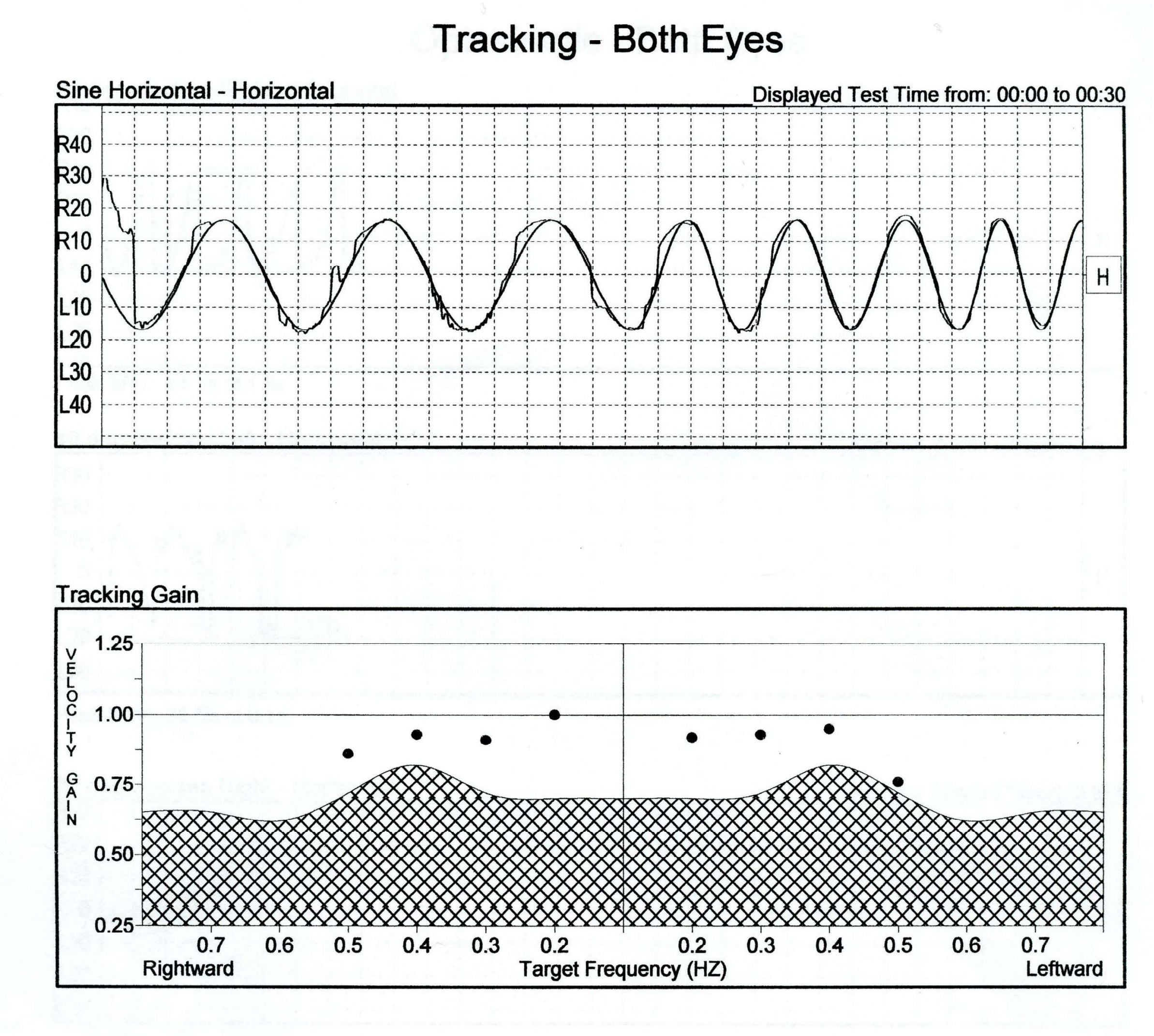

Supplementary Figure 2. Tracking result on both eyes tested on Day 76, demonstrating an intense decreasing in accuracy.
