# Supplementary material for: Intratympanic steroid treatments rescued recurrent hearing loss following COVID-19 vaccination and detection of an intralabyrinthine schwannoma
Source: BMJ Case Rep. 2022 Jul 6;15(7):e249316. doi: 10.1136/bcr-2022-249316 (PMC9260791; doi:10.1136/bcr-2022-249316)
Supplement: Supplementary data [file bcr-2022-249316supp004.pdf]

Supplementary Figure 4: SARS COV-2 TOTAL ANTIBODY

Component Results

Component	Your Value	Standard Range
**SARS-CoV-2 Total Antibody, anti-spike protein**	**Your Value**<br>**Reactive**	*Standard Range*<br>*Nonreactive*

Test performed using the Siemens Advia Centaur SARS-CoV-2 Total assay. This result obtained using the FDA Emergency Use Authorization (EUA) test from Siemens. Nonreactive results do not rule out SARS-CoV-2 infection, particularly in those who have been in very recent contact with the virus. (Follow-up testing with a molecular diagnostic should be considered to rule out infection in such individuals.) Results from antibody testing should not be used as the sole basis to diagnose or exclude SARS-CoV-2 infection, or to inform infection status.Reactive results may be due to past or present infection with non-SARS-CoV-2 coronavirus strains, such as coronavirus HKU1, NL63, OC43, or 229E. The performance of this assay has not been established with cord blood, neonatal specimens, or body fluids other than serum or plasma. Not for the screening of donated blood. Index values < 1.0 are considered nonreactive; index values > or = to 1.0 are considered reactive."

**SARS-COV-2 Total Index, anti-spike protein**	**Your Value**<br>**>10.00** Index	*Standard Range*<br>*<=1.00 Index*

MyChart® licensed from Epic Systems Corporation © 1999 - 2020
